# Supplementary material for: From sequence to enzyme mechanism using multi-label machine learning
Source: BMC Bioinformatics. 2014 May 19;15:150. doi: 10.1186/1471-2105-15-150 (PMC4229970; doi:10.1186/1471-2105-15-150)
Supplement: Additional file 2 — Java code of ml2db. Additional file ml2db_code.tar.gz contains the Java source code to run the multi-label machine learning experiments and save the results to database. The code’s Javadoc is included. [file 1471-2105-15-150-S2.zip › additional file 2/ml2db/utils/doc/test/XmlNodeTest.html]

XmlNodeTest


---


|  |  |  |
| --- | --- | --- |
| **Overview** \| **Package** \| **Class** \| **Use** \| **Tree** \| **Deprecated** \| **Index** \| **Help** | |  |
| **PREV CLASS**   **NEXT CLASS** | **FRAMES**    **NO FRAMES**     **All Classes** |  |
| SUMMARY: NESTED \| FIELD \| CONSTR \| METHOD | DETAIL: FIELD \| CONSTR \| METHOD |  |


---


## test Class XmlNodeTest

```
java.lang.Object
  junit.framework.Assert
      junit.framework.TestCase
          test.XmlNodeTest
```

**All Implemented Interfaces:**: junit.framework.Test

---

``` public class XmlNodeTest extends junit.framework.TestCase ```

Class

**Version:**
:   18 Nov 2008

**Author:**
:   Luna De Ferrari luna.deferrari-at-ed.ac.uk

---

**Constructor Summary**

`XmlNodeTest()`


| **Method Summary** | |
| --- | --- |
| `static XmlNode` | `getNumberedXmlTree()` |
| `static XmlNode` | `getPdbSimpleElement()` |
| `static java.lang.String` | `getXml1String()` |
| `static java.lang.String` | `getXml2String()` |
| `static java.lang.String` | `getXml3String()` |
| `static java.lang.String` | `getXml4String()` |
| `static java.lang.String` | `getXml5String()` |
| `static java.lang.String` | `getXml6String()` |
| `static java.lang.String` | `getXml7String()` |
| `static java.lang.String` | `getXmlString()` |
| `void` | `setUp()` |
| `void` | `testAddChildElement()` |
| `void` | `testNodeHeadToString()` |
| `void` | `testSimpleElement()` |
| `void` | `testToString()` |

**Methods inherited from class junit.framework.TestCase**

`countTestCases, getName, run, run, runBare, setName, toString`

**Methods inherited from class junit.framework.Assert**

`assertEquals, assertEquals, assertEquals, assertEquals, assertEquals, assertEquals, assertEquals, assertEquals, assertEquals, assertEquals, assertEquals, assertEquals, assertEquals, assertEquals, assertEquals, assertEquals, assertEquals, assertEquals, assertEquals, assertEquals, assertFalse, assertFalse, assertNotNull, assertNotNull, assertNotSame, assertNotSame, assertNull, assertNull, assertSame, assertSame, assertTrue, assertTrue, fail, fail, failNotEquals, failNotSame, failSame, format`

**Methods inherited from class java.lang.Object**

`equals, getClass, hashCode, notify, notifyAll, wait, wait, wait`

**Constructor Detail**

### XmlNodeTest

```
public XmlNodeTest()
```


**Method Detail**

### setUp

```
public void setUp()
           throws java.lang.Exception
```

:   **Overrides:**: `setUp` in class `junit.framework.TestCase`
:   **Throws:**: `java.lang.Exception`

---


### testAddChildElement

```
public void testAddChildElement()
```

---


### testNodeHeadToString

```
public void testNodeHeadToString()
```

---


### testSimpleElement

```
public void testSimpleElement()
```

---


### testToString

```
public void testToString()
```

---


### getNumberedXmlTree

```
public static XmlNode getNumberedXmlTree()
```

---


### getPdbSimpleElement

```
public static XmlNode getPdbSimpleElement()
```

:   **Returns:**: an xml node containing PDB data

---


### getXml1String

```
public static java.lang.String getXml1String()
```

---


### getXml2String

```
public static java.lang.String getXml2String()
```

---


### getXml3String

```
public static java.lang.String getXml3String()
```

---


### getXml4String

```
public static java.lang.String getXml4String()
```

---


### getXml5String

```
public static java.lang.String getXml5String()
```

---


### getXml6String

```
public static java.lang.String getXml6String()
```

---


### getXml7String

```
public static java.lang.String getXml7String()
```

---


### getXmlString

```
public static java.lang.String getXmlString()
```


---


|  |  |  |
| --- | --- | --- |
| **Overview** \| **Package** \| **Class** \| **Use** \| **Tree** \| **Deprecated** \| **Index** \| **Help** | |  |
| **PREV CLASS**   **NEXT CLASS** | **FRAMES**    **NO FRAMES**     **All Classes** |  |
| SUMMARY: NESTED \| FIELD \| CONSTR \| METHOD | DETAIL: FIELD \| CONSTR \| METHOD |  |


---
